# A hierarchical deep learning approach with transparency and interpretability based on small samples for glaucoma diagnosis

**DOI:** 10.1038/s41746-021-00417-4

**Published:** 2021-03-11

**Authors:** Yongli Xu, Man Hu, Hanruo Liu, Hao Yang, Huaizhou Wang, Shuai Lu, Tianwei Liang, Xiaoxing Li, Mai Xu, Liu Li, Huiqi Li, Xin Ji, Zhijun Wang, Li Li, Robert N. Weinreb, Ningli Wang

**Affiliations:** 1 Department of Mathematics, Beijing University of Chemical Technology, Beijing, China; 2 National Key Discipline of Pediatrics, Ministry of Education, Department of Ophthalmology, Beijing Children’s Hospital, Capital Medical University, Beijing, China; 3 Beijing Institute of Ophthalmology, Beijing Tongren Hospital, Capital Medical University, Beijing Ophthalmology & Visual Science Key Lab, Beijing, China; 4 School of Information and Electronics, Beijing Institute of Technology, Beijing, China; 5 School of Electronic and Information Engineering, Beihang University, Beijing, China; 6 Beijing Shanggong Medical Technology co., Ltd, Beijing, China; 7 Shiley Eye Institute, University of California San Diego, La Jolla, CA USA; 8 Beijing Advanced Innovation Center for Big Data-Based Precision Medicine, Beihang University & Capital Medical University, Beijing Tongren Hospital, Beijing, China

**Keywords:** Glaucoma, Computer science

## Abstract

The application of deep learning algorithms for medical diagnosis in the real world faces challenges with transparency and interpretability. The labeling of large-scale samples leads to costly investment in developing deep learning algorithms. The application of human prior knowledge is an effective way to solve these problems. Previously, we developed a deep learning system for glaucoma diagnosis based on a large number of samples that had high sensitivity and specificity. However, it is a black box and the specific analytic methods cannot be elucidated. Here, we establish a hierarchical deep learning system based on a small number of samples that comprehensively simulates the diagnostic thinking of human experts. This system can extract the anatomical characteristics of the fundus images, including the optic disc, optic cup, and appearance of the retinal nerve fiber layer to realize automatic diagnosis of glaucoma. In addition, this system is transparent and interpretable, and the intermediate process of prediction can be visualized. Applying this system to three validation datasets of fundus images, we demonstrate performance comparable to that of human experts in diagnosing glaucoma. Moreover, it markedly improves the diagnostic accuracy of ophthalmologists. This system may expedite the screening and diagnosis of glaucoma, resulting in improved clinical outcomes.

## Introduction

Disease diagnosis technology based on artificial intelligence (AI), especially deep neural networks (DNN), is rapidly improving. Multiple medical studies use AI to make automatic diagnosis and achieve accuracy nearly as high as experts^1–7^. However, real world clinical implementation of AI technology is not yet widespread. The lack of transparency and interpretability of AI algorithms is a major obstacle to the application of AI algorithms in clinical practice^8^. Enhancing the understanding or interpretation of how a given AI technology achieves certain decisions or predictions might enhance its clinical implementation^9^. It is thought that if the AI algorithm’s reasoning cannot be explained, then physicians cannot verify whether the reasoning is plausible^10^. Therefore, many physicians seek transparency and interpretability of AI algorithms to facilitate patient safety.

Instead of a single physician’s mistake harming a patient, the potential for an AI algorithm inducing iatrogenic risk is vast. Therefore, the use of AI algorithms as a tool to assist physicians in diagnosis, rather than giving a completely independent diagnosis, is desirable^11^. Moreover, before AI systems are applied to clinical diagnosis, one should verify and evaluate the actual influence of adjunctive AI systems on physicians’ diagnostic abilities.

In addition, most DNN models are limited by their need for tens of thousands of well-labeled samples^1–7^ for training. In the case of supervised learning, the accuracy of the DNN algorithm prediction relies heavily on the accuracy of the underlying annotations inputted into the algorithms; poorly labeled data will yield poor results^8,12^. Only medical experts can give high-quality sample annotations, and such large-scale sample annotations will inevitably be laborious and costly. Therefore, designing AI algorithms based on small samples with high accuracy is an important direction of current AI research.

For AI diagnosis of ophthalmic diseases based on fundus images, considerable progress has been made in recent years. DNN algorithms are designed to diagnose diseases such as diabetic retinopathy and age-related macular degeneration based on fundus images^1,13–16^. In addition, the DNN models are also used to predict cardiovascular risk factors based on fundus images^17^. In these studies, the DNN algorithms are black-box models. Although these models have achieved high enough prediction accuracy, the prediction results cannot be understood by physicians.

In the field of AI diagnosis of glaucoma based on fundus images, satisfactory prediction accuracy has been obtained. Ting et al. designed a deep learning (DL) system for identifying multiple eye diseases including glaucoma in multiethnic populations with diabetes^15^. This system was trained for detecting possible glaucoma using 125,189 fundus images with an AUC of 0.942. Li et al. developed a DL system for detecting glaucoma based on 31,745 fundus photographs from various clinical settings, with an AUC of 0.986^18^. Liu et al. established a DL system for detection of glaucoma using a total of 281,943 fundus photographs and assessed its generalization ability in various data sets, reporting an AUC of 0.996 in primary local validation dataset^19^. In above three researches, the training of DL systems requires tens of thousands of training samples, which has led to costly investment in developing such AI diagnostic systems. In order to alleviate the dependence on large training samples, Christopher et al. applied transfer learning and data augmentation technology to establish a DL system for glaucoma screening based on 14,822 training samples, and obtained an AUC of 0.91^20^. The design of above DL systems does not use expert knowledge, but rather only mines statistical information, which leads to the need for large training samples. In addition, the diagnostic logic of the DL systems in above researches is not transparent to physicians, and the diagnostic results are not interpretable. When making glaucoma diagnosis based on fundus images, glaucoma specialists need to synthesize a variety of features, such as vertical cup-to-disk ratio (VCDR), shape of neuroretinal rim, hemorrhage, peripapillary atrophy, retinal nerve fiber layer defects (RNFLD), and so on. Integration of a physician’s diagnostic reasoning into the design of the AI would make the results of the diagnostic system easier to understand and accept, while reducing the requirement for training sample capacity.

In this study, we developed a hierarchical deep learning system (HDLS) using 1791 fundus photographs for glaucoma diagnosis. Compared with the studies mentioned above, this research has made the following technical novelties: First, the diagnostic thinking of glaucoma experts is integrated into the design of DNN, and high prediction accuracy is obtained using only small training samples. Second, a framework that integrates deep classification network and deep segmentation network is designed, and the classification network is used to assist in improving the segmentation accuracy of optic cup (OC) by the segmentation network. Considering that OC is the most important anatomical structure in glaucoma diagnosis, the improvement of OC segmentation accuracy further improves the accuracy of glaucoma diagnosis. Last, this integrated system is transparent and interpretable: In addition to providing automatic diagnosis results, this system can also provide diagnosis logic and diagnosis confidence assessment. Above technical novelty provides a practical solution of AI for assisted diagnosis of glaucoma in the real world.

## Results

### Overall Data

We developed a HDLS using 1791 fundus photographs from Beijing Tongren Hospital. We evaluated the predictive performance of this system on validation dataset 1 and validation dataset 2. Validation dataset 1 includes 6301 fundus photographs from Beijing Tongren Hospital; Validation dataset 2 includes 1964 fundus photographs from two hospitals in Tibet and Ningxia Autonomous Region in China. Furthermore, we evaluated the accuracy of doctors’ diagnosis with the assistance of HDLS on validation dataset 3, which includes 400 fundus photographs randomly selected from the validation dataset 1. The demographic information of the training and validation datasets is shown in Table 1.

**Table 1:** Baseline characteristics of participants.

Datasets	Source Datasets	No			Age Mean (SD), y	Female No./Total (%)	Cohort	Ethnicity/Race,	Assessor
		Images	Eyes	Individuals					
Training dataset	Tongren	1791	1791	975	52.6 (13.5)	532 (54.6)	Clinic-based	Han-Chinese	2 senior Glaucoma specialists
Validation dataset 1	Tongren	6301	6301	3371	51.9 (14.6)	1675 (49.7)	Clinic-based	Han-Chinese	2 senior Glaucoma specialists
Validation dataset 2	Tibet and Ningxia	1964	1964	1027	53.8 (15.2)	533 (51.9)	Clinic-based	Tibetan-Chinese Hui-Chinese Han-Chinese	2 senior Glaucoma specialists
Validation dataset 3	Tongren	400	400	386	54.3 (11.5)	186 (48.2)	Clinic-based	Han-Chinese	2 senior Glaucoma specialists

The 1791 fundus photographs in training dataset included 875 images with referable glaucomatous optic neuropathy (GON) and 916 images with unlikely GON. To train the image segmentation network, the optic disc (OD), OC, and RNFLD of the fundus images in training dataset were manually labeled by one senior glaucoma specialist (M.H.). The 6301 fundus photographs in validation dataset 1 included 2884 images with referable GON and 3417 images with unlikely GON. The 1964 fundus photographs in validation dataset 2 included 619 images with referable GON and 1345 images with unlikely GON. Validation dataset 3 included 200 images with referable GON and 200 images with unlikely GON.

### Study pipeline

**Fig. 1:**
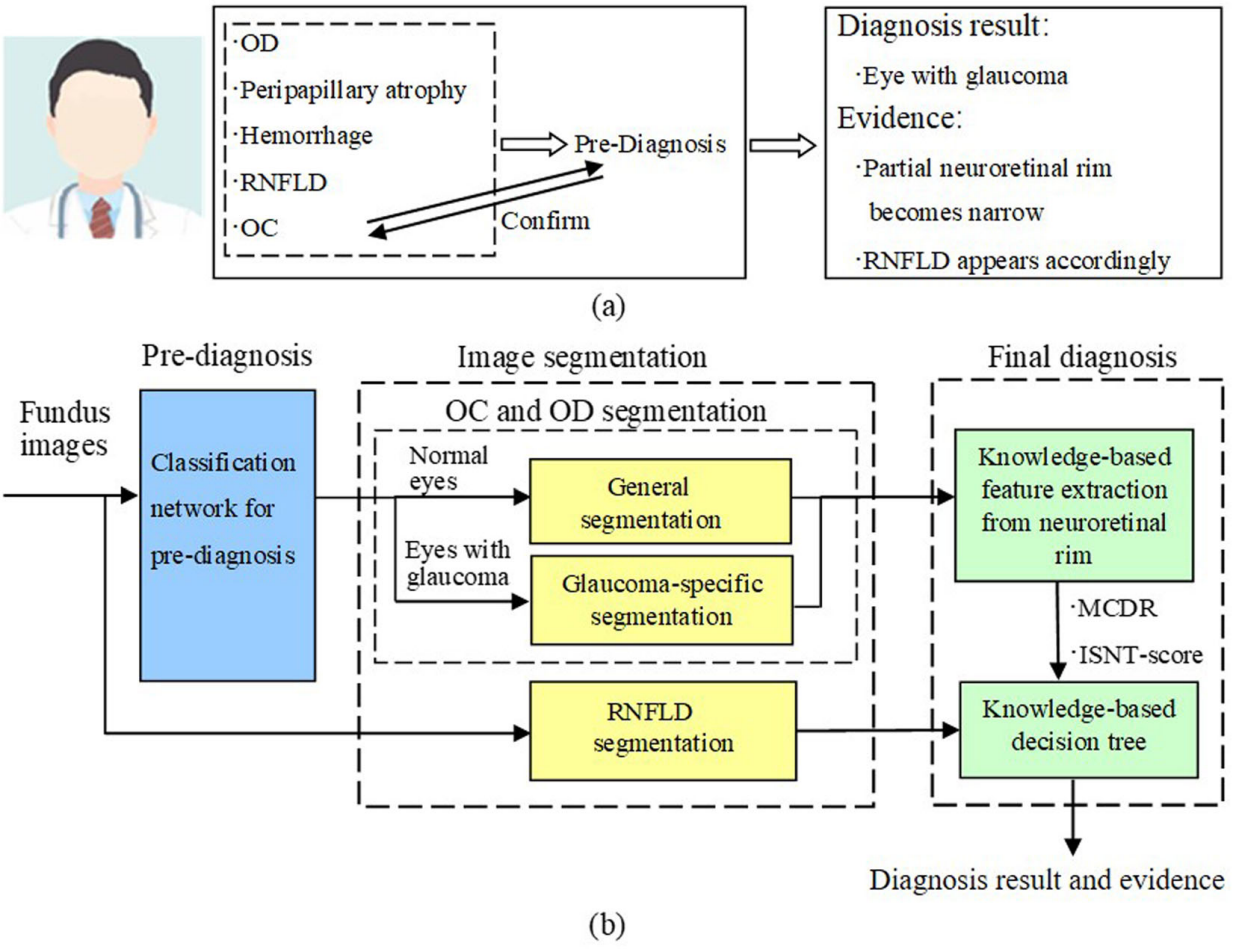
Schematic diagram of the hierarchical deep learning system (HDLS) simulating doctors’ diagnostic thinking. **a** The process of doctors’ diagnosis of glaucoma. **b** The process of diagnosing glaucoma of the HDLS. This system includes three modules: The pre-diagnosis module is used for preliminary diagnosis based on the overall information of the fundus photographs. The image segmentation module is used to segment OD, OC, and RNFLD based on the pre-diagnostic results. For fundus photographs pre-diagnosed as glaucoma, a glaucoma-specific network is used to segment the OC. The final diagnosis module is used to calculate MCDR and ISNT-score based on the segmentation. Finally, these two indicators are used to make a final diagnosis in combination with the status of RNFLD. OD optic disc, OC optic cup, RNFLD retinal nerve fiber layer defects, MCDR mean cup-to-disk ratio, ISNT inferior, superior, nasal, temporal.

The proposed HDLS comprehensively simulates the diagnostic thinking of glaucoma experts. In Fig. 1 (a), it is showed how an ophthalmologist diagnoses glaucoma. The position of the OD is determined, and whether there is peripapillary atrophy, hemorrhage, or RNFLD is ascertained. The topography of the OC is evaluated and this information is combined with the above to determine whether there is glaucomatous optic neuropathy. In fact, the determination of the OC and the diagnosis of glaucoma is an interactive process. In Fig. 1 (b), it is showed how the HDLS simulates the diagnostic thinking of glaucoma experts. This system includes three modules: pre-diagnosis module, image segmentation module, and final diagnosis module. The pre-diagnosis module is used for preliminary diagnosis based on the overall information of the fundus photographs. The image segmentation module is used to segment image features based on the preliminary diagnostic results. In particular, for fundus photographs pre-diagnosed as glaucoma, a glaucoma-specific network is used to segment the OC. The final diagnosis module is used to analyze the characteristics of the neuroretinal rim based on the segmentation. In this process, the mean cup-to-disk ratio (MCDR) is used to describe the scale characteristics, and the ISNT (inferior, superior, nasal, temporal) score is used to describe the morphological characteristics of the rim. Finally, these two indicators are used to make a final diagnosis in combination with the status of RNFLD.

### Transparency and interpretability of the model

**Fig. 2:**
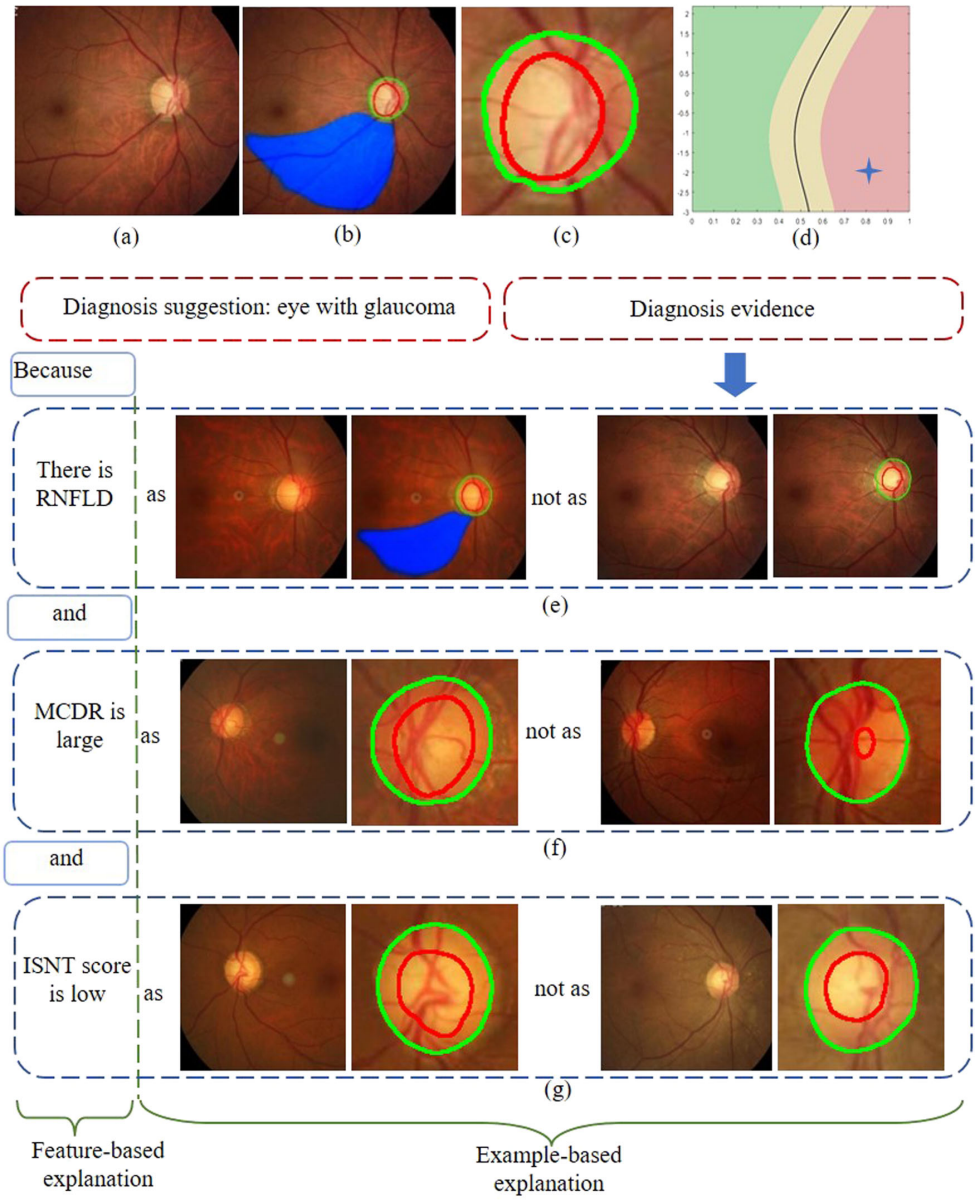
Schematic of transparency and interpretability for hierarchical deep learning system. **a** Original fundus image. **b** Segmentation of OD, OC, and RNFLD. **c** Magnification of the image shown in (**b**). **d** Two-dimensional plane of MCDR and ISNT score. **e**–**g** Comparison of the fundus image currently being evaluated with fundus images that have been clearly diagnosed in the database in terms of MCDR, ISNT score, and RNFLD. OD optic disc, OC optic cup, RNFLD retinal nerve fiber layer defects, MCDR mean cup-to-disk ratio, ISNT inferior, superior, nasal, temporal.

The HDLS gives diagnosis evidence in a form that ophthalmologists can understand. The diagnostic evidence consists of three parts, as shown in Fig. 2. The first part is the segmentation of OD, OC, and RNFLD (see Fig. 2b and c), the second part is a two-dimensional feature display: the MCDR and ISNT score based on image segmentation (see Fig. 2d), and the third part is the comparison of the fundus image currently being evaluated with fundus images that have been clearly diagnosed in the database, in terms of MCDR, ISNT score, and RNFLD (see Fig. 2e–g). Furthermore, the HDLS can display a predictive reliability. In a two-dimensional plane composed of the MCDR and ISNT score, the red region indicates a diagnosis of glaucoma with high confidence, green indicates a diagnosis of normal eye with high confidence, and yellow indicates a diagnosis with low confidence.

### Performance of the HDLS in validation datasets 1 and 2

**Fig. 3:**
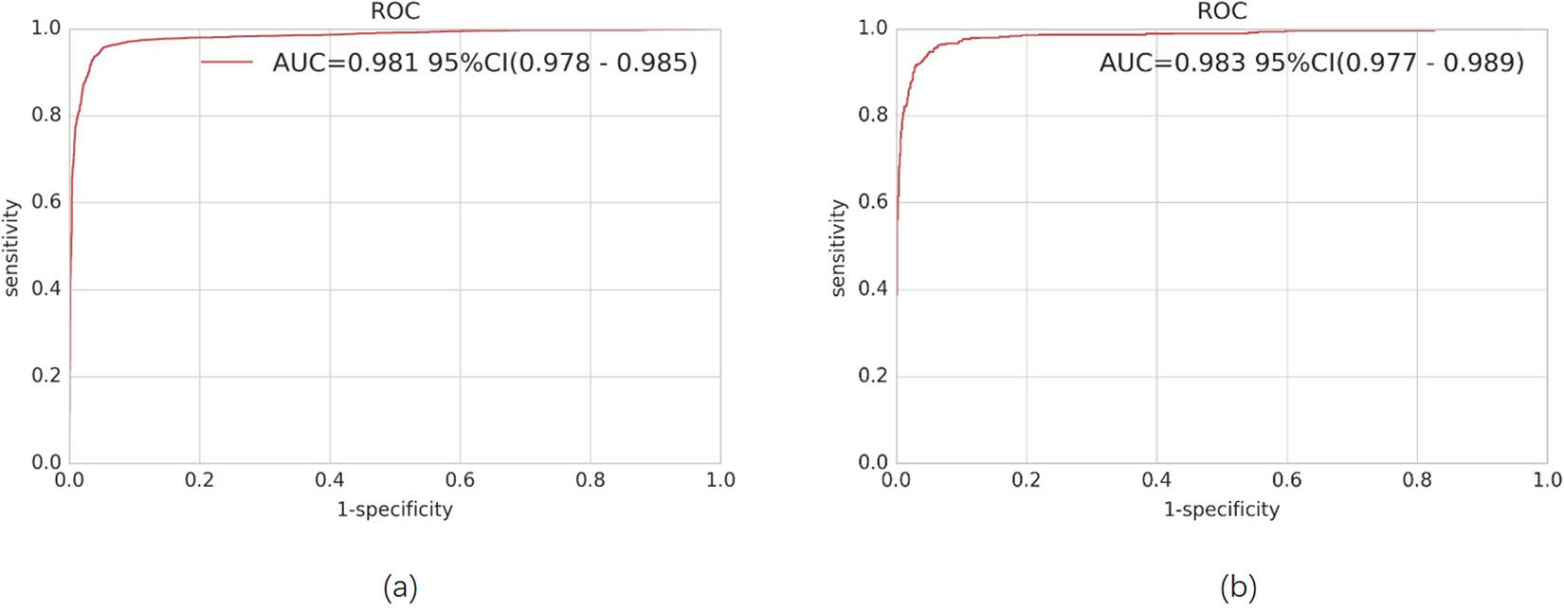
Graph showing receiver operating characteristic (ROC) curve derived from the validation dataset 1. **a** and validation dataset 2 (**b**) that was obtained using the hierarchical deep learning system. AUC area under curve.

In validation dataset 1, we evaluated the performance of the proposed HDLS. Figure 3 shows the receiver operating characteristic (ROC) curve. We can see that, the area under the ROC curve (AUC) is 0.981 (95% CI, 0.978-0.985). From Table 2 we can see that, when the threshold is set as 0.8, the sensitivity and specificity reach 96.1% (95% CI, 95.3–96.8%) and 93.9% (95% CI, 93.1–94.7%), respectively. Furthermore, we evaluate the performance of the HDLS in reliable region and suspicious region, respectively. The sensitivity and specificity in the reliable region can reach 97.7% (95% CI, 97.0–98.3%) and 97.8% (95% CI, 97.2–98.4%), which are significantly higher than the results in the suspicious region. However, the sensitivity and specificity in the suspicious region can still reach 91.9% (95% CI, 90.0–93.8%) and 83.2% (95% CI, 80.8–85.7%).

**Table 2:** Performance of the hierarchical deep learning system in different confidence regions on validation datasets 1 and 2.

		Number of images				% (95% CI)	
Datasets	Regions	True	False	True	False	Sensitivity	Specificity
		Positive	Negative	Negative	Positive		
	Overall region	2770	114	3210	207	0.9605 (0.953-0.968)	0.9394 (0.931-0.947)
validation dataset 1	Reliable region	2019	48	2455	55	0.9768 (0.970-0.983)	0.9781 (0.972-0.984)
	Suspicious region	751	66	755	152	0.9192 (0.900-0.938)	0.8324 (0.808-0.857)
	Overall region	592	27	1265	80	0.9564 (0.940-0.973)	0.9405 (0.928-0.953)
validation dataset 2	Reliable region	493	8	929	17	0.9840 (0.973-0.995)	0.9820 (0.974-0.991)
	Suspicious region	99	19	336	63	0.8390 (0.772-0.906)	0.8421 (0.806-0.878)

In validation dataset 2, the AUC is 0.983 (95% CI, 0.977–0.989), and when the threshold is set as 0.8, the sensitivity and specificity reaches 95.6% (95% CI, 94.0–97.3%) and 94.1% (95% CI, 92.8–95.3%), respectively. Furthermore, the sensitivity and specificity in the reliable region are significantly higher than the results in the suspicious region.

We compared the prediction accuracy of the proposed HDLS with two automatic glaucoma diagnosis algorithms based on deep learning^19,21^. In validation dataset 1 and 2, the proposed HDLS achieved the highest AUC values. The detailed experimental results were showed in Supplementary Fig. 1 in Supplementary Note 1.

### Accuracy of OD, OC, and RNFLD segmentation by HDLS

We presented the segmented OD, OC, and RNFLD by HDLS in validation dataset 3 to two glaucoma experts (M.H. and H.W.). The two glaucoma experts independently classify the segmentation into completely correct, approximately correct, and incorrect (Table 3). The sum of completely correct and approximately correct for OD, OC, and RNFLD segmentation reached 99.75%, 97.25% and 97.50%, respectively. We further analyzed the reasons of incorrect segmentation. The 2 cases of incorrect segmentation of OD are due to the influence of peripapillary atrophy. And 15 out of 22 cases of incorrect segmentation of OC are due to the tilt of the OD. In fundus images with referable GON, 10 out of 12 cases of missing segmentation of RNFLD (false negative) are due to diffuse RNFLD; and in fundus images with unlikely GON, the 8 cases of incorrect segmentation of RNFLD (false positive) are due to the uneven distribution of the optic nerve fiber layer.

**Table 3:** Accuracy evaluation of hierarchical deep learning system for OD, OC, and RNFLD segmentation.

Segmentation	Images with referable GON			Images with unlikely GON		
	Completely-correct No. (%)	Approximately correct No. (%)	Incorrect No. (%)	Completely-correct No. (%)	Approximately correct No. (%)	Incorrect No. (%)
OD	386 (96.5)	12 (3)	2 (0.5)	391 (97.75)	9 (2.25)	0 (0)
OC	331(82.75)	53 (13.25)	16 (4)	380 (95)	14 (3.5)	6 (1.5)
RNFLD	372 (93)	16 (4)	12 (3)	392 (98)	0 (0)	8 (2)

*OD* optic disc, *OC* optic cup, *RNFLD* retinal nerve fiber layer defects, *GON* glaucomatous optic neuropathy.

### Efficacy of MCDR and ISNT score in diagnosing glaucoma

**Fig. 4:**
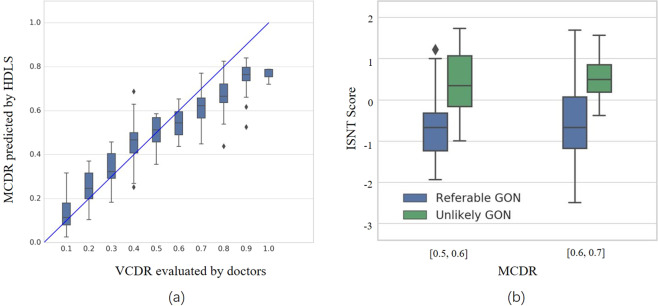
Evaluation of efficacy of MCDR and ISNT score. **a** Comparison of MCDR predicted by hierarchical deep learning system (HDLS) with VCDR evaluated by the glaucoma expert. **b** Comparison of ISNT score obtained by HDLS between referable GON and unlikely GON. The median (50th percentile) is represented by the horizontal centerline, and the 25th and 75th percentiles as the lower and upper limits of the box. The upper and lower horizontal bars represent the maximum and minimum values, respectively, of a 1.5 interquartile range. The diamond symbols represent outliers. Outliers were denoted in the figure because they resided outside of the 1.5 interquartile range. MCDR mean cup-to-disk ratio, VCDR vertical cup-disc ratio, ISNT inferior, superior, nasal, temporal; GON glaucomatous optic neuropathy.

Based on the segmentation of OD and OC, we calculated two indicators, the MCDR and ISNT score. The MCDR is compared with the VCDR evaluated by a glaucoma expert (M.H.). From Fig. 4 (a) it can be seen that, the median of the MCDR obtained by HDLS is roughly equivalent to the VCDR by the glaucoma expert. The correlation coefficient value of VCDR estimated by the glaucoma expert and MCDR predicted by HDLS is 0.93 (*P* < 0.001). We also compared the efficacy of HDLS with general network and glaucoma-specific network and found that the mean absolute error of the MCDR predicted by the HDLS is smaller than both the general network and glaucoma-specific network (Supplementary Fig. 2 in Supplementary Note 2). From Fig. 4 (b), we can see that, in case the MCDR predicted by HDLS is in the range [0.5, 0.6] and [0.6, 0.7], compared with eyes with referable GON, eyes without GON have higher ISNT scores (*P* < 0.001 and *P* = 0.014, respectively). This indicates that the ISNT score has a significant effect on distinguishing referable GON from Non-GON.

### Accuracy of diagnosing glaucoma by doctors with the assistance of HDLS

**Fig. 5:**
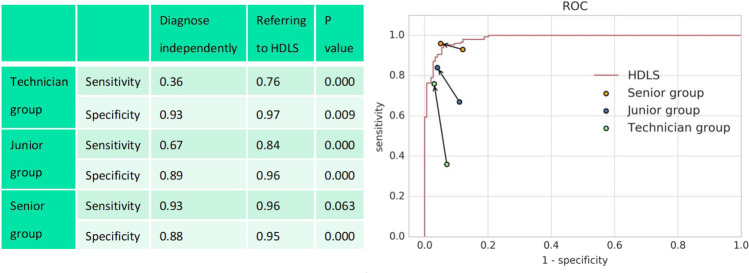
Comparison between doctors’ independent diagnosis and doctors’ diagnosis with the assistance of hierarchical deep learning system (HDLS). In the right figure, the starting points of the arrows represent the results of independent diagnosis, and the end points represent the results with the assistance of HDLS. The red line represents the receiver operating characteristic (ROC) curve of the HDLS.

We tested the accuracy of diagnosing glaucoma by doctors with the assistance of HDLS in validation dataset 3. Four senior ophthalmologists, four junior ophthalmologists, and four technicians were invited to participate in the evaluation. It can be seen from Fig. 5 that the mean sensitivity and specificity of independent diagnosis of the junior group and technician group is significantly lower than that of the HDLS; the mean sensitivity and specificity of independent diagnosis of the senior group is roughly equivalent to that of the HDLS. It is notable that both the junior group and technician group significantly improved the sensitivity and specificity when referring to the diagnosis recommendations of HDLS (*P* < 0.01). The senior group also improved the sensitivity and specificity when referring to the diagnostic recommendations of the HDLS (*P* = 0.063 and *P* < 0.001, respectively), and the average diagnostic accuracy exceeded that of HDLS.

**Fig. 6:**
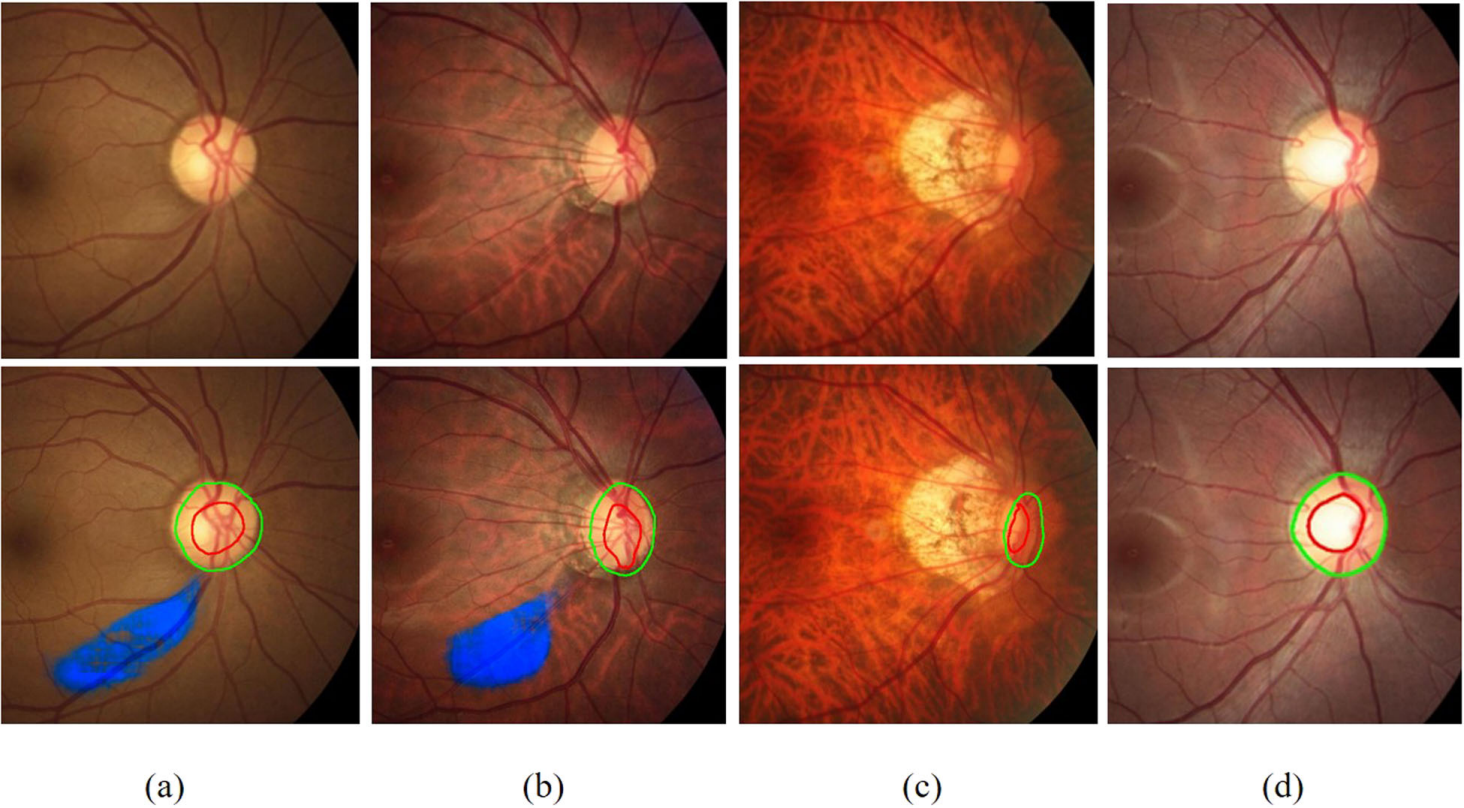
Examples of fundus images misdiagnosed by junior doctors and technicians. Above: original images; Below: images analyzed by hierarchical deep learning system (HDLS). **a** Glaucomatous fundus image with insignificant retinal nerve fiber layer defects (RNFLD). **b** Glaucomatous fundus image with vascular curvature information indicating localized rim thinning. **c** Glaucomatous fundus image with high myopia. **d** Normal fundus image with physiologic large cup.

On the validation dataset 3, each of the four junior ophthalmologists independently diagnosed 100 fundus images with referable GON and 100 fundus images with unlikely GON. The three most common reasons for missed diagnosis of glaucoma were: (1) not identifying insignificant RNFLD, (2) using only color but not vascular curvature information to identify localized rim thinning, (3) difficulty in assessing highly myopic eyes with severe tilt of the disc. For 90 out of 104 cases of these situations, HDLS can provide clues to correct diagnosis. The most common reason for the misclassification of normal eyes to glaucoma for junior ophthalmologists is physiologic large cups. For 28 out of 35 cases of this situation, HDLS can calculate a high ISNT score, indicating that the rim conforms to the ISNT rule, thus giving a clue that the eye tends to be normal. (Fig. 6).

## Discussion

Recently, several reports of deep learning methods for detecting glaucoma from retinal fundus images with high sensitivity and specificity have been published^15,18–20^. However, these models are black box models. In clinical reality, doctors cannot understand the mechanism of the models’ prediction, and therefore it is difficult for them to adopt the results. In addition, the training of these models often requires tens of thousands of well-labeled samples, which requires huge costs. Our study has a number of unique differences when compared with the previous studies. First, we integrate the deep classification network and the deep segmentation network into one system, which not only considers the global information of the image, but also fuses the prior knowledge of the experts to achieve a sufficiently high prediction accuracy (AUC of 0.981, sensitivity of 96.1%and specificity of 93.9% on validation dataset 1; AUC of 0.983, sensitivity of 95.6% and specificity of 94.1% on validation dataset 2) using a small training dataset. Second, this system not only gives the diagnosis results, but also provides a diagnosis basis consistent with the expert’s thinking, so that it can be understood and accepted by ophthalmologists. It also provides important prerequisites for clinical implementation. Finally, we evaluated the practicality of the system in the clinic. We find that when doctors use this system, the diagnostic accuracy is significantly improved, which shows application prospect of this system. In China, there are a large number of areas with scarce medical resources and a shortage of glaucoma specialists, such as the Tibet Autonomous Region and Ningxia Hui Autonomous Region. The proposed HDLS achieved high prediction accuracy for glaucoma diagnosis on the fundus photographs collected from the above two regions (validation dataset 2), which provided a powerful tool for AI-aided glaucoma diagnosis in these regions.

Several DNN-based OC and OD features extraction models for glaucoma detection have been proposed. Liu et al.^19^ used DNN for glaucoma diagnosis and a heat map showed that the features were extracted from the OD area, but how DNN used these features to make a diagnosis was incomprehensible to doctors. Fu et al.^21^ used DNN to segment the OD and OC. The VCDR was calculated and used as an indicator to diagnose glaucoma. However, only using VCDR to diagnose glaucoma is far from fully simulating doctors’ complete diagnosis idea. We compared the accuracy of the proposed HDLS with the two methods mentioned above in diagnosing glaucoma. In both validation dataset 1 and 2, the proposed HDLS achieved the highest AUC values.

In the field of deep learning, ensemble learning is an important method to improve the accuracy of DNN prediction^22^. Most existing ensemble learning frameworks have a parallel structure: the prediction results of multiple DNNs determine the final prediction^23^. In this study, we proposed a HDLS, which used multiple decision-making modules to jointly determine the final prediction in series. In this system, the pre-diagnosis module is at the front, the image segmentation module is at the middle, and the feature extraction and final decision-making module is at the end. The output of the image segmentation module is transparent, and the feature extraction and final decision modules are transparent and interpretable. The integrated framework design of this series structure has the following advantages: on the one hand, it integrates the prediction performance of each DNN, which improves the accuracy of prediction, on the other hand, the final prediction of the system is transparent and interpretable, and it is easier for doctors to understand and accept.

This system simulates an ophthalmologist’s reasoning to diagnose glaucoma. The decision-making process of HDLS designed in this study is a process of mutual confirmation of diagnosis and segmentation. When making a diagnosis, various features of fundus images need to be integrated. Among them, the segmentation of the OC is a key and difficult point. For some fundus images, it is difficult to reflect the three-dimensional structure of the OC with limited two-dimensional information, which makes it even difficult for doctors to segment the OC accurately. However, the thinning of the rim is often accompanied by the corresponding position of RNFLD or splinter hemorrhage and these features provide hints and clues to the determination of the OC. The precise segmentation of the OC provides an important basis for the final diagnosis of glaucoma. In the HDLS, the prediction of the pre-diagnosis module is based on the global information of the image and provides clues for the segmentation of the OC. For the images pre-diagnosed as glaucoma, a segmentation network for suspected glaucoma is applied; for the images pre-diagnosed as normal, a general segmentation network is applied. The idea of designing an integrated diagnostic system by simulating the reasoning of an ophthalmologist is also relevant for intelligent diagnosis design of other diseases.

The output of the segmentation module is the boundaries of the OD, OC, and RNFLD. The further feature extraction module calculates MCDR and ISNT score based on the OD and OC boundaries. These results can be visualized on the fundus images (see Fig. 2b, c), which is convenient to evaluate the accuracy of segmentation based on one’s own experience. The MCDR and ISNT score can also be displayed on a two-dimensional plane (see Fig. 2d), so that doctors can have a quantitative understanding of the glaucomatous features in the image. And the final diagnosis is made based on the status of MCDR, ISNT score and RNFLD. Based on each index, the image to be evaluated are compared with those with clear diagnosis in the database (see Fig. 2e–g), and the diagnosis is explained based on the examples. In addition, HDLS also gives confidence of prediction. For samples located in reliable regions (see red and green regions in Fig. 2d), the sensitivity and specificity reached 97.7% and 97.8% in validation dataset 1, and 98.4% and 98.2% in validation dataset 2, respectively. The division of the confidence region provides an important reference for doctors to make a final diagnosis based on their own experiences and HDLS’s recommendations.

Despite the promising results, our study has several limitations. First, the diagnostic evidence of HDLS did not include other factors besides OC, OD, and RNFLD, such as hemorrhage and peripapillary atrophy. Although the black-box classification network of the pre-diagnostic module may incorporate these factors, it is not explicitly expressed in the final diagnosis result. In addition, every fundus image was resized to a resolution of 512×512 given the limitations in the GPU’s computational power. As a result, the fine texture details of the retinal fiber layer are partially lost in the compressed fundus images, which affected the accuracy of the segmentation module in detecting RNFLD. Especially for diffuse RNFLD in glaucoma, the prediction of the segmentation module has false negatives; for normal fundus images with uneven retina fiber layer thickness, the prediction of the segmentation module has false positives. Furthermore, the proposed HDLS was only evaluated in the medical records collected in the ophthalmology clinic, but not on the general population records. Considering that glaucoma-assisted screening at medical examination centers is an important potential application scenario of HDLS, it is of great significance to evaluate our model in natural populations.

## Methods

### Ethics approval

The study was conducted according to the tenets of the Declaration of Helsinki and it was approved by the institutional review board (IRB) of Beijing Tongren Hospital (identifier, 121 2017BJTR519). As the study was a retrospective review and analysis of fully anonymized color retinal fundus images, the medical ethics committee exempted the need for the patients’ informed consent.

### Study participants and fundus photograph grading

To establish the HDLS, a total of 2000 fundus images were obtained from Beijing Tongren hospital from 2013 to 2014. For patients with same name or ID number, only one of them was included. The 2000 fundus images include 1000 glaucoma and 1000 non-glaucoma based on the initial label when imported into the database which matches the most recent diagnosis of the patient. For the initial glaucoma diagnosis, clinicians comprehensively evaluated the fundus image, visual field, and intraocular pressure. Glaucoma was defined as eyes with either glaucomatous optic neuropathy (GON) or glaucomatous visual field loss, with or without raised intraocular pressure. GON was defined as either diffuse or focal narrowed neuroretinal rim; excavation; RNFL defects; peripapillary hemorrhages; or cup-disc ratio ≥0.7. Glaucomatous visual field loss was diagnosed if any of the following findings were evident on two consecutive visual field tests: a glaucoma hemifield test outside normal limits, pattern standard deviation (PSD) <5%, or a cluster of three or more nonedge points in typical glaucomatous locations, all depressed on the pattern deviation plot at a level of *P* < 0.05, with one point in the cluster depressed at a level of *P* < 0.01. Then each image in the training dataset was subjected to a 2- tiered grading system. The label was masked to evaluators. The first tier of graders consisted of 1 trained medical student and 1 nonmedical undergraduate. They conducted initial quality control according to the following criteria which has been described previously^19^: (1) the image did not contain severe resolution reductions or significant artifacts; (2) the image field included the entire optic nerve head and macula; (3) the illumination was acceptable (ie, not too dark or too light); (4) the image was focused sufficiently for grading the optic nerve head and retinal nerve fiber layer. The second tier of graders consisted of 2 senior glaucoma specialists with more than 10 years of experience with glaucoma diagnosis (M.H. and H.W.). This study focuses on using deep learning to predict glaucomatous structural changes in fundus images. Therefore, the two glaucoma experts used only the fundus image to detect GON, without reference to intraocular pressure or visual field. Each grader independently diagnosed and recorded each image according to the criteria in Table 4. After this process, images were classified as unlikely, probable, and definite GON. Referable GON was defined as probable or definite GON. There were 140 images graded as poor quality and 69 images of which the two glaucoma specialists were disagreed on the diagnosis. These images were excluded from the training dataset. To train the image segmentation network, the OD, OC, and RNFLD of the remaining fundus images were manually labeled by one senior glaucoma specialist (M.H.).

**Table 4:** Classification for glaucomatous optic neuropathy.

Classification	Clinical features
Unlikely glaucomatous optic neuropathy	No sign of the conditions below
Probable glaucomatous optic neuropathy	Any conditions of the following:
	0.7 ≤ VCDR < 0.85;
	general rim thinning ≥60° or localized rim
	thinning <60° (11 to 1 o´clock or 5 to 7 o´clock);
	RNFL defects;
	peripapillary hemorrhages.
Definite Glaucomatous optic neuropathy	Any conditions of the following:
	VCDR ≥ 0.85;
	localized notches;
	RNFL defect corresponds to narrowing of rim or localized notches.

*RNFL* retinal nerve fiber layer, *VCDR* vertical cup-disc ratio.

To verify the repeatability of the labeling results, M.H. labeled the boundaries of the OD, OC, and RNFLD in the training dataset twice (the interval between the two markings is more than one month). We used Dice coefficient as an index to evaluate the consistency of two labeling results. Dice coefficient is defined as the ratio of the intersection area and average area of two labeling results. On the training set, the average Dice coefficient of the OD, OC and RNFLD labeled twice by M.H. were 0.97, 0.91 and 0.90, respectively. This shows that the labeling results of H.M. have high repeatability. To verify the reliability of the labeling results, another senior glaucoma specialist (H.W.) also labeled the boundaries of the OD, OC, and RNFLD in the training dataset. The average Dice coefficient of the OD, OC and RNFLD labeled by H.W. and M.H. in the first time was 0.96, 0.87, and 0.88, respectively. This shows that the labeling results of the two senior glaucoma specialists are highly consistent. Finally, we used the OD, OC, and RNFLD labeled by M.H. in the first time as the ground truth of the training dataset.

To test the HDLS, we collected fundus images from multiple centers: A total of 7000 fundus images were obtained from Beijing Tongren hospital from 2015 to 2018 and a total of 2100 fundus images were obtained from two hospitals in western China (People’s Hospital of Ningxia Hui Autonomous Region, Tibetan Hospital of Tibet Autonomous Region) from 2012 to 2018. For patients with same name or ID number, we only included one of them. To avoid overlap between validation and training datasets, patients with the same name or ID number as those in the training dataset were excluded. The 7000 fundus images in validation dataset 1 include 3300 glaucoma and 3700 non-glaucoma based on the initial label when imported into the database which matches the most recent diagnosis of the patient. Then each image was also subjected to a 2- tiered grading system mentioned above. There were 475 images graded as poor quality and 224 images of which the two glaucoma specialists were disagreed on the diagnosis. These images were excluded. The remaining images constitute the validation dataset 1.

The 2100 fundus images in validation dataset 2 include 700 glaucoma and 1400 non-glaucoma based on the initial label when imported into the database which matches the most recent diagnosis of the patient. Then each image was also subjected to a 2- tiered grading system mentioned above. There were 82 images graded as poor quality and 54 images of which the two glaucoma specialists were disagreed on the diagnosis. These images were excluded. The remaining images constitute the validation dataset 2.

From validation dataset 1, we randomly sampled 200 fundus images with referable GON and 200 fundus images with unlikely GON to constitute validation dataset 3. On validation dataset 3, we evaluated the accuracy of HDLS in extracting the image features (OD, OC, and RNFLD), the statistical significance of the MCDR and ISNT scores predicted by HDLS for diagnosing glaucoma. At last we tested the accuracy of doctors’ prediction when diagnosing glaucoma assisted by HDLS. Three groups of doctors were invited to participant in the test including 4 senior doctors (ophthalmologists with more than 8 years of work experience), 4 junior doctors (ophthalmologists with 3–5 years of work experience), and 4 technicians (non-ophthalmologists temporarily trained for glaucoma diagnosis). To make testing fairer, we randomly selected 100 fundus images with referable GON and 100 fundus images with unlikely GON to form dataset A, and the remaining cases formed dataset B. The first (last) two doctors of each group made diagnosis on fundus images in dataset A (B) independently, and made diagnosis on fundus images in dataset B (A) with reference to HDLS.

### Model development

The proposed HDLS includes three modules: pre-diagnosis, image (OD, OC and RNFLD) segmentation, and final diagnosis based on expert knowledge. Among them, the first two modules are designed based on deep learning. A DNN model is a sequence of mathematical operations applied to the input, such as pixel values in an image. There can be millions of parameters in this mathematical function^24^. Deep learning is the process of training the parameters of the DNN to perform a given task.

In this study, the pre-diagnosis was treated as a classification problem in deep learning: predict whether a fundus image is with GON. We used the Inception-v3 neural-network architecture^25^ to make the pre-diagnosis. The segmentation of OD, OC, and RNFLD is treated as an image segmentation problem in deep learning: predict whether each pixel belongs to the target region. We used a U-shaped convolutional DNN^26^ to make the image segmentation. For the OD segmentation, during the training process, the parameters of the DNN are initially set to random values. Then, for each annotated fundus image, the OD segmented by the DNN is compared with that annotated by the glaucoma expert, and the parameters of the DNN are then modified slightly to decrease the error on that image (stochastic gradient descent). This process is repeated for every image in the training set many times over. Finally, the locations of OD segmented by the DNN will be very close to those annotated by glaucoma specialist for images in the training set. The training process of DNNs for glaucoma pre-diagnosis, OC segmentation and RNFLD segmentation is like that of OD segmentation.

In the pre-diagnosis module, based on the above-mentioned deep learning methods, a pre-diagnosis classification network (PDCN) is built using the fundus images in training dataset. This PDCN is a black-box predictor and focuses on the global information of a fundus image to make glaucoma pre-diagnosis. This network is used to simulate the doctor’s initial diagnosis based on the global information of a fundus image.

In the image segmentation module, for the segmentation of OD and RNFLD, the annotated locations of OD and RNFLD in training dataset are used to train a segmentation network, respectively. For the segmentation of OC, we design two different networks: A general segmentation network (GSN) and a glaucoma-specific segmentation network (GSSN). First, the annotated location of OC in all fundus images in training dataset is used to train the GSN. And then, the annotated location of OC in glaucoma fundus images in training dataset is used to train the GSSN. In the prediction of fundus images in the validation set, for fundus images predicted as normal eyes by the PDCN, the OC is segmented using the GSN; and for the fundus images predicted with glaucoma by the PDCN, the OC is segmented using the GSSN.

In the final diagnosis module, firstly, the segmentation of OD and OC are used to extract features based on expert knowledge. The MCDR and ISNT score are computed based on the OD and OC segmentation as quantitative indicators reflecting the features of the neuroretinal rim. MCDR is the mean CDR within a range of 360 degrees along the neuroretinal rim; ISNT score is a quantitative indicator of the ISNT rule^27^. ISNT rule describes the special asymmetric structure of the rim of typical normal eyes: the inferior rim is the widest portion of the rim, followed by the superior rim, the nasal rim, and the temporal rim. If the CDR of a typical normal eye in the range of 0-360 degrees is displayed in a Cartesian coordinate system (the horizontal axis represents the position, and the vertical axis represents the value of the CDR) and fits it as a curve, then this curve generally has a typical bimodal structure. The curve of glaucoma generally does not have this specific structure. Therefore, we designed the ISNT score as an indicator to describe the degree of matching between the rim of the fundus image to be predicted and the above-mentioned specific structure: a large ISNT score indicates that the rim conforms to the ISNT rule. Considering that for two standardized curves with similar shapes, their corresponding vectors have a large inner product, we designed the method to calculate ISNT score as follows.

First, a CDR vector with 360 dimensions is computed based on the segmentation of OD and OC. Second, the CDR vector is normalized: each component is subtracted from the average of this vector and divided by the standard deviation of this vector. Finally, the inner product of this normalized vector and a baseline vector is calculated as the ISNT score. Here, the baseline vector is the average CDR vector of 500 typical normal eyes. The fundus images of these typical normal eyes were collected in the physical examination center of Tongren Hospital, and are not included in the training dataset and validation datasets. The OD and OC of each fundus image are labeled by a senior glaucoma specialist (M.H.).

Furthermore, based on the MCDR and ISNT score mentioned above, a two-dimensional classification line is established by support vector machines (SVM)^28^. The final diagnostic criteria are with a decision tree^29^ structure: (1) if there is a RNFLD, then it is predicted as glaucoma; (2) if there is no RNFLD, then make a prediction based on the two-dimensional classification line: (i) cases above or on the classification line are predicted as normal eyes, (ii) cases below the classification line is predicted as glaucoma.

In the above prediction process, the pre-diagnosis of the classification network has a certain deviation, leading to the prediction of some normal eyes as glaucoma, so that while the sensitivity of the final diagnosis is improved, the specificity is slightly reduced, resulting in an imbalance between sensitivity and specificity in the final diagnosis. Compared with threshold of 0.5, the threshold of 0.8 for the softmax output makes the specificity of the pre-diagnosis classification network higher than the sensitivity, thereby reducing the imbalance between the sensitivity and specificity of the final diagnosis. Therefore, the threshold was set as 0.8 for the softmax output of the classification network. In all the following evaluations on validation datasets, the threshold was kept as 0.8.

### Evaluating the algorithm

On validation datasets 1 and 2, we used sensitivity, specificity and AUC to evaluate the performance of the proposed HDLS. For the AUC of HDLS, it needs to be calculated based on the probability p that HDLS predicts as GON for each sample. In this study, this probability is calculated based on the RNFLD state predicted by the segmentation module and the probability p_1_ of glaucoma predicted by the SVM based on the MCDR and ISNT score: For fundus images with RNFLD, set p = (1 + p_1_) / 2; for fundus images without RNFLD, set p = p_1_.

On validation dataset 3, two glaucoma experts evaluate the accuracy of HDLS for segmenting OC, OD, RNFLD according to the following criteria: If there is no deviation in the segmentation of the target area, it is determined to be completely correct; if there is a deviation in the segmentation of the target area but the deviation does not affect the diagnosis result, it is determined to be approximately correct; if the deviation in the segmentation of the target area is too large to affect the diagnosis result, it is determined to be incorrect. And then, we used the correlation coefficient between the MCDR predicted by HDLS and VCDR marked by the glaucoma expert to evaluate the accuracy of HDLS for predicting VCDR. On validation dataset 3, we used sensitivity and specificity to evaluate the accuracy of doctors to diagnose glaucoma independently and with the assistance of the HDLS.

### Statistical analysis

The 95% CIs for AUC were calculated non-parametrically through logit-transformation-based CIs. In addition to AUC, the sensitivity and specificity of each operating point in ROC curves were also measured with 2-sided 95% CIs. These CIs were calculated as Clopper-Pearson intervals, which are exact intervals based on cumulative probabilities. In comparing the ISNT scores of glaucoma and normal eyes, a Wilcoxon rank-sum test was used. The chi-squared independence test was used when assessing the increased sensitivity and specificity of doctors when referenced HDLS diagnosis over independent diagnosis. All statistical tests were performed using SPSS (Statistical Product and Service Solutions) software.

### Reporting summary

Further information on research design is available in the Nature Research Reporting Summary linked to this article.

## Supplementary information

Supplementary Information

Reporting Summary

## Data Availability

The data analyzed during the study are available from the corresponding author upon reasonable request.

## Code availability

The codes used to perform the study are available from the corresponding author upon reasonable request.
