# Supplementary material for: A hierarchical deep learning approach with transparency and interpretability based on small samples for glaucoma diagnosis
Source: NPJ Digit Med. 2021 Mar 11;4:48. doi: 10.1038/s41746-021-00417-4 (PMC7952384; doi:10.1038/s41746-021-00417-4)
Supplement: Supplementary file 1 — Supplementary Information [file 41746_2021_417_MOESM1_ESM.pdf]

Supplementary Note 1

We used the training dataset in this study to train the ResNet[21] and M-Net[23], and the experimental results were reported in Supplementary Figure 1. As can be seen from this figure, the proposed hierarchical deep learning system (HDLS) achieved the highest AUC values in both validation dataset 1 and 2. Moreover, compared with the M-Net[23], the proposed HDLS had significant advantages.

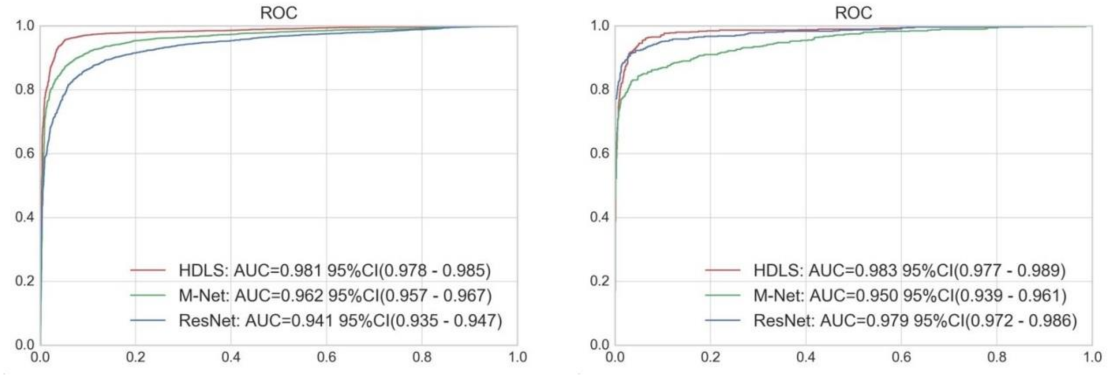

Supplementary Figure 1. Graphs showing ROC curves derived from the validation dataset 1 (left) and validation dataset 2 (right) that were obtained using HDLS, M-Net and ResNet.

Abbreviations: ROC, receiver operating characteristic; HDLS, hierarchical deep learning system; AUC, area under curve;

Supplementary Note 2

In validation dataset 3, we compared the accuracy of the cup-to-disk ratio (CDR) calculated based on the general network, glaucoma-specific network and proposed hierarchical deep learning network. The box plots of the three methods to predict CDR were reported in Supplementary Figure 2. As can be seen from this figure, the MCDR calculated based on the glaucoma-specific network tends to be larger than that of the general network. Therefore, while reducing false negatives, the glaucoma-specific network may increase false positives. For the case of real VCDR ⩾0.5, the prediction of the glaucoma-specific network was closer to the ground truth than general network; for the case of real VCDR⩽0.4, the prediction of general network was closer to the ground truth than the glaucoma-specific network. In the case of real VCDR⩽0.3, the prediction of the proposed hierarchical deep learning network was closer to that of the general network; when the real VCDR⩾0.5, it was closer to the prediction of the glaucoma-specific network. In validation set 3, the mean absolute errors of the MCDR predicted by the general network, glaucoma-specific network and hierarchical deep learning network was 0.11, 0.11 and 0.09, respectively.

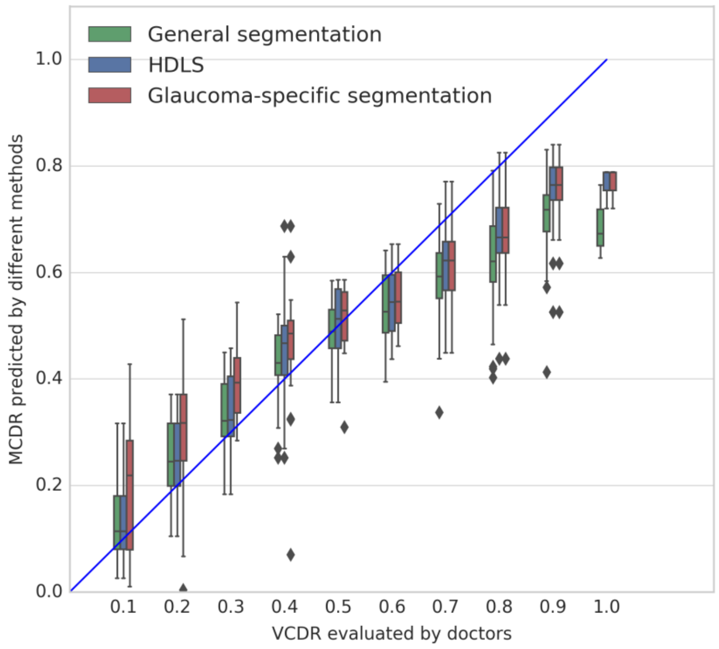

Supplementary Figure 2. Comparison of MCDR predicted by different methods with VCDR evaluated by the glaucoma expert.

Abbreviations: HDLS, hierarchical deep learning system
