# Supplementary material for: A hierarchical deep learning approach with transparency and interpretability based on small samples for glaucoma diagnosis
Source: NPJ Digit Med. 2021 Mar 11;4:48. doi: 10.1038/s41746-021-00417-4 (PMC7952384; doi:10.1038/s41746-021-00417-4)
Supplement: Supplementary file 2 — Reporting Summary [file 41746_2021_417_MOESM2_ESM.pdf]

nature research

Corresponding author(s): Ningli Wang, Li Li

Last updated by author(s): Sep 8, 2020

Reporting Summary

Nature Research wishes to improve the reproducibility of the work that we publish. This form provides structure for consistency and transparency in reporting. For further information on Nature Research policies, see our Editorial Policies and the Editorial Policy Checklist.

Statistics

For all statistical analyses, confirm that the following items are present in the figure legend, table legend, main text, or Methods section.

n/a	Confirmed	
☐	☒	The exact sample size (*n*) for each experimental group/condition, given as a discrete number and unit of measurement
☒	☐	A statement on whether measurements were taken from distinct samples or whether the same sample was measured repeatedly
☐	☒	The statistical test(s) used AND whether they are one- or two-sided<br>*Only common tests should be described solely by name; describe more complex techniques in the Methods section.*
☒	☐	A description of all covariates tested
☒	☐	A description of any assumptions or corrections, such as tests of normality and adjustment for multiple comparisons
☐	☒	A full description of the statistical parameters including central tendency (e.g. means) or other basic estimates (e.g. regression coefficient) AND variation (e.g. standard deviation) or associated estimates of uncertainty (e.g. confidence intervals)
☐	☒	For null hypothesis testing, the test statistic (e.g. *F*, *t*, *r*) with confidence intervals, effect sizes, degrees of freedom and *P* value noted<br>*Give P values as exact values whenever suitable.*
☒	☐	For Bayesian analysis, information on the choice of priors and Markov chain Monte Carlo settings
☒	☐	For hierarchical and complex designs, identification of the appropriate level for tests and full reporting of outcomes
☐	☒	Estimates of effect sizes (e.g. Cohen's *d*, Pearson's *r*), indicating how they were calculated

Our web collection on statistics for biologists contains articles on many of the points above.

Software and code

Policy information about availability of computer code

Data collection	No software was used.
Data analysis	All statistical tests were performed using SPSS (Statistical Product and Service Solutions) software.

For manuscripts utilizing custom algorithms or software that are central to the research but not yet described in published literature, software must be made available to editors and reviewers. We strongly encourage code deposition in a community repository (e.g. GitHub). See the Nature Research guidelines for submitting code & software for further information.

Data

Policy information about availability of data

All manuscripts must include a data availability statement. This statement should provide the following information, where applicable:

- Accession codes, unique identifiers, or web links for publicly available datasets
- A list of figures that have associated raw data
- A description of any restrictions on data availability

The data analyzed during the study are available from the corresponding author upon reasonable request.

Field-specific reporting

Please select the one below that is the best fit for your research. If you are not sure, read the appropriate sections before making your selection.

☒ Life sciences ☐ Behavioural & social sciences ☐ Ecological, evolutionary & environmental sciences

For a reference copy of the document with all sections, see nature.com/documents/nr-reporting-summary-flat.pdf

Life sciences study design

All studies must disclose on these points even when the disclosure is negative.

Sample size	In this study, we collected 8265 fundus photos as a verification set. The sample size of the validation set is large enough to exceed that in most similar studies.
Data exclusions	In training dataset, there were 140 images graded as poor quality and 69 images of which the two glaucoma specialists were disagreed on the diagnosis. These images were excluded from the training dataset. In validation dataset 1, there were 475 images graded as poor quality and 224 images of which the two glaucoma specialists were disagreed on the diagnosis. These images were excluded from validation dataset 1. In validation dataset 2, there were 82 images graded as poor quality and 54 images of which the two glaucoma specialists were disagreed on the diagnosis. These images were excluded from validation dataset 2.
Replication	In multiple repeated experiments, the prediction difference of AUC does not exceed 1% is taken as the measure of repeatability. In this study, all attempts of replication were successful.
Randomization	We have selected consecutive cases of glaucoma and non-glaucoma eyes in a period of time. Therefore, the selection of samples is random.
Blinding	Two glaucoma experts used only the fundus image to detect GON, without reference to intraocular pressure, visual field and original diagnosis result.

Reporting for specific materials, systems and methods

We require information from authors about some types of materials, experimental systems and methods used in many studies. Here, indicate whether each material, system or method listed is relevant to your study. If you are not sure if a list item applies to your research, read the appropriate section before selecting a response.

Materials & experimental systems

n/a	Involved in the study	
☒	☐	Antibodies
☒	☐	Eukaryotic cell lines
☒	☐	Palaeontology and archaeology
☒	☐	Animals and other organisms
☐	☒	Human research participants
☒	☐	Clinical data
☒	☐	Dual use research of concern

Methods

n/a	Involved in the study	
☒	☐	ChIP-seq
☒	☐	Flow cytometry
☒	☐	MRI-based neuroimaging

Human research participants

Policy information about studies involving human research participants

Population characteristics	We gave a detailed information in Table 1 in the manuscript, as follows: In training dataset, mean age was 52.6±13.5 years， and 54.6% were female. In validation dataset 1, mean age was 51.9±14.6 years, and 49.7% were female. In validation dataset 2, mean age was 53.8±15.2 years, and 51.9% were female. The 1791 fundus photographs in training dataset included 875 images with referable glaucomatous optic neuropathy (GON) and 916 images with unlikely GON. The 6301 fundus photographs in validation dataset 1 included 2877 images with referable GON and 3417 images with unlikely GON. The 1964 fundus photographs in validation dataset 2 included 619 images with referable GON and 1345 images with unlikely GON. Validation dataset 3 included 200 images with referable GON and 200 images with unlikely GON.
Recruitment	Firstly, the retinal images of patients in this study are consecutively recruited; Secondly, to avoid overlap, for patients with same name or ID number, we only included one of them; Thirdly, each image in the study was subjected to a 2- tiered grading system for quality control and labeling. The initial diagnosis of the image was masked to evaluators. The above 3 processes ensure that there is no bias in the recruitment of cases.
Ethics oversight	The study was conducted according to the tenets of the Declaration of Helsinki and it was approved by the institutional review board (IRB) of Beijing Tongren Hospital (identifier, 121 2017BJTR519). As the study was a retrospective review and

analysis of fully anonymized colour retinal fundus images, the medical ethics committee exempted the need for the patients' informed consent.

Note that full information on the approval of the study protocol must also be provided in the manuscript.
